# Mitochondrial Dysfunction Links Ceramide Activated HRK Expression and Cell Death

**DOI:** 10.1371/journal.pone.0018137

**Published:** 2011-03-31

**Authors:** Farhan Rizvi, Tom Heimann, Anja Herrnreiter, William J. O'Brien

**Affiliations:** 1 Department of Ophthalmology, Medical College of Wisconsin, Milwaukee, Wisconsin, United States of America; 2 Department of Microbiology/Molecular Genetics, Medical College of Wisconsin, Milwaukee, Wisconsin, United States of America; Vanderbilt University Medical Center, United States of America

## Abstract

**Purpose:**

Cell death is an essential process in normal development and homeostasis. In eyes, corneal epithelial injury leads to the death of cells in underlying stroma, an event believed to initiate corneal wound healing. The molecular basis of wound induced corneal stromal cell death is not understood in detail. Studies of others have indicated that ceramide may play significant role in stromal cell death following LASIK surgery. We have undertaken the present study to investigate the mechanism of death induced by C6 ceramide in cultures of human corneal stromal (HCSF) fibroblasts.

**Methods:**

Cultures of HCSF were established from freshly excised corneas. Cell death was induced in low passage (p<4) cultures of HCSF by treating the cells with C6 ceramide or C6 dihydroceramide as a control. Cell death was assessed by Live/Dead cell staining with calcein AM and ethidium homodimer-1 as well as Annexin V staining, caspase activation and TUNEL staining Mitochondrial dysfunction was assessed by Mito Sox Red, JC-1 and cytochrome C release Gene expression was examined by qPCR and western blotting.

**Results:**

Our data demonstrate ceramide caused mitochondrial dysfunction as evident from reduced MTT staining, cyto *c release* from mitochondria, enhanced generation of ROS, and loss in mitochondrial membrane potential (ΔΨ_m_). Cell death was evident from Live -Dead Cell staining and the inability to reestablish cultures from detached cells. Ceramide induced the expression of the harikari gene(HRK) and up-regulated JNK phosphorylation. In ceramide treated cells HRK was translocated to mitochondria, where it was found to interact with mitochondrial protein p32. The data also demonstrated HRK, p32 and BAD interaction. Ceramide-induced expression of HRK, mitochondrial dysfunction and cell death were reduced by HRK knockdown with HRK siRNA.

**Conclusion:**

Our data document that ceramide is capable of inducing death of corneal stromal fibroblasts through the induction of HRK mediated mitochondria dysfunction.

## Introduction

Mitochondria are the "power house" of the cell and as such they are organelles that are critically involved in pathways of cell death. In response to molecular cues from death stimuli, mitochondria release molecules known as apoptosis inducing factors, cytochrome c (cyto *c*) and generate reactive oxygen species (ROS) in resulting cell death [1], [2]. The mitochondrial outer membrane permeabilization (MOMP) is a critical factor in mitochondrial mediated cell death. In this process proteins sequestered in the outer and inner mitochondrial membranes are permitted to interact with the proteins of cytosol resulting in conformational changes thus leading to demise of the cell [3]. MOMP is regulated by several classes of proteins that include non Bcl2 members, Bcl-2 family members, and a subset of BH3 only proteins that share BCl-2 homology domain3 [4], [5], [6]. It has been proposed that BH3-only proteins influence the MOMP through directly binding and inactivating their specific anti-death Bcl2 partners. On the other hand according to a "hierarchy model" certain BH3-only proteins may bypass the direct binding to pro or anti death proteins by acting upstream or downstream of anti-death factors [6], [7], [8]. Thus, BH3 only proteins can cause the mitochondrial dysfunction either dependent or independent of pro-death factors BAX and BAK [6], [8]. BH3-only protein activities can be regulated by several ways in initiating the different signals that ultimately converge on the mitochondria causing its dysfunction and thereby the cell death [9]. Ceramide has been associated with the regulation of Bcl-2 family as well as BH3 only proteins [10].

Ceramide, the structural backbone of sphingolipids/sphingomyelin, is an important second messenger involved in cell growth, differentiation, and death [11]. The specific mechanism(s) by which ceramide regulates BH3-only protein activities relating to mitochondrial dysfunction and cell death is not understood in detail. Studies have documented detrimental effects of ceramide on the mitochondrial integrity and functions including: generation of reactive oxygen species, ATP depletion, collapse in the inner mitochondrial membrane potential, inhibition and/or activation of the activities of various components of the mitochondrial electron transport chain and release of inter-membrane space (IMS) proteins [11], [12]. Mitochondrial dysfunction has been implicated in the death of corneal fibroblasts and corneal epithelial cells as evident by the release of cyto *c*, loss in mitochondrial membrane potential (ΔΨ_m_), and ROS release [13], [14].

Corneal epithelial injury, as a result of keratorefractive surgeries such as LASIK; lead to stromal cell death is a critical event in corneal wound healing [15]. Ceramide has been hypothesized to been involved in the response of stromal cells to injury [16]. Ceramide can cause the death of rabbit corneal stromal fibroblast grown in culture but the mechanism of cell death was not determined [16]. BH3-only proteins such as BAD and HRK are known to be regulated by ceramide in causing death of non-ocular cell types. Ceramide induced dephosphorylation of BAD in HepG2 [12] and Cos7 cells [17]. It also induced the expression of HRK in oligodendrocytes [18]. Dephosphorylated BAD translocate to mitochondria, bypassing BAX and BAK switch [12], whereas HRK directly interacted with the mitochondrial protein p32, a protein known for its role in causing mitochondrial dysfunction and cell death [19], [20]. Thus, both BAD and HRK are capable of causing mitochondrial dysfunction by directly acting with mitochondrial proteins, independent of BAX and BAK.

The studies presented here investigate the molecular basis of corneal stromal cell death induced by C6 ceramide. We examined mitochondrial dysfunction and determined the role of BH3 only protein, HRK, in mediating death. The key findings of the present study are that C6 ceramide cause the release of cyto *c* from mitochondria, generation of ROS, and collapse of ΔΨ_m_ documenting mitochondrial dysfunction. Ceramide caused rapid transient JNK phosphorylation leading to enhanced expression of *HRK*. HRK was translocated to mitochondria of ceramide treated cells where it interacted with BAD and mitochondrial p32. These observations suggest that BAD and HRK as BH3 only proteins are directly involved in causing mitochondrial dysfunction [12], [19].

## Materials and Methods

### Cell culture

Human corneal stromal cells were isolated and grown as fibroblasts (HCSF) from human donor eyes purchased from Wisconsin's Lion Eye Bank as previously described [21]. Briefly, after removal of the corneal epithelium and endothelium the stroma was digested for 16 h at 37°C with collagenase (Clostridium histolyticium, Invitrogen, Carlsbad, CA) in Hank's balanced salts solution (HBSS) containing penicillin G and streptomycin sulfate (both from Sigma Aldrich, St. Louis, MO). Cells were recovered by centrifugation, suspended in growth medium, and grown at 34°C in Dulbecco's Modified Eagle Medium (DMEM) (Invitrogen),supplemented with 5% heat-inactivated defined fetal bovine serum (FBS; Hyclone/ Thermo Scientific, Waltham, MA), 0.1% Mito+serum extender (BD Biosciences, San Jose, CA), and 10 µg/ml ciprofloxacin (Sigma Aldrich). Cells of the first four passages were used for these studies. In the experiments where fluorescent microscopy or fluorometry was performed cells were incubated in DMEM without phenol red (31053, Invitrogen) while other components remained the same.

### Reagents

C_6_ ceramide (ceramide) and C_6_ dihydroceramide (dihydroceramide); (BIOMOL International, Plymouth Meeting, PA) were dissolved in DMSO (Sigma Aldrich, St Louis, MO) and used at concentration of 0- 40 µM, SP600125 (Sigma Aldrich, St. Louis, MO) was used at concentration of 10 µM. Antibodies included; anti-HRK (AHP1178, AbD Serotec, Oxford, UK), anti-Cytochrome c (SC-8385), anti-P32/C1QBP(SC-10258), anti-BAD(SC-81442 Santa Cruz Biotechnology, Santa Cruz, CA); anti JNK-p/SAPK-p ( 9251) and anti-JNK/SAPK(9252); Cell Signaling, Danver, MA), β-actin (A5316), β-tubulin (T5201), SigmaAldrich, St. Louis, MO). HRK ON-Target Plus siRNA pool (siHRK) and non target control (siNTC) were purchased from Dharmacon (Lafayette, CO). We determine the optimum concentration of siRNA to be 40 nM concentration.

### Live and Dead Cell Assay

HCSF were incubated for up to16 h in the presence or absence of ceramide. Preliminary dose response assays confirmed that 40 µM ceramide was the ED50 for HCSF death. Cells were treated for 1 h in the dark with 4 mM calcein-AM and 2 mM ethidium homodimer-I (Eth-D1) prepared in 1XHBSS with Ca and Mg. Live/dead cell measurement was done according to the manufactures instructions (LIVE/DEAD Viability and Cytotoxicity Kit) (Molecular Probes, Invitrogen).

### MTT Assay

HCSF were plated at a density of about 1×10^5^ cells per cm^2^ in DMEM containing 5% FBS, Mito+ and ciprofloxacin. After 24 to 48 hrs the media was changed to DMEM containing 5 % FBS without phenol red supplemented with 40 µM ceramide or dihydroceramide. Growth and survival of HCSF was measured based on the reduction of MTT [3-(4, 5-dimethylthiazol-2-yl)-2, 5-diphenyltetrazolium bromide. Briefly, 900 µl serum free medium and 100 µl MTT solution (5 mg/ml in Ca2+ and Mg2+ free PBS, (SigmaAldrich, St. Louis, MO) were added to each, well containing cells and incubated at 34°C for 1 h-2 h. The insoluble formazan was extracted with 1.0 ml isopropanol solution (containing 0.04 M HCL; Fisher Scientific, USA), 1 ml per well, at 34°C for additional 15 min. The extracting liquid and cells were harvested and centrifuged at 5,000× G for 10 min. The amount of formazan in the supernatant was estimated by reading the absorbance at 650 and 570. Three replicates were read for each sample, the mean value was used as the final result.

### Assessment of the Mitochondrial Membrane Potential

Changes in the mitochondrial membrane potential (ΔΨ_m_) were measured using JC-1 dye (JC100, Cell Technology, Mountain View, CA). Briefly, HCSF were seeded in at 70-80% confluent levels and treated with 40 µM C6 ceramide or C6 dihydroceramide in triplicates for 16 h. Cells along with floaters were pooled, centrifuged ( 500 xg /5 min), washed with HBSS, resuspended with JC-1 (1∶200) in Hanks buffer with Ca^2+^ and Mg^2+^, and were incubated (37°C; 30 minutes). Cells were then centrifuged (500 xg/5 min) and washed at least three times with HBSS to remove the excess probe. 100 µl of resuspended cell sample was used to read the fluorescence in 96 wells assay clear bottom, black plate (Costar 3603, Corning NY, USA) at Ex 498/ Em 535 and Ex 560/ Em590 in FLX800 micro plate fluorescent reader (Bio-Tek Instrument). The ratio of red: green was calculated as Δψ_m_. For fluorescent microscopy cells were also counterstained with 5 µl/10 ml Hoechst 33342 (H3570, Invitrogen, Madison, WI) in HBSS containing Ca^2+^ and Mg^2+^ and the images were captured.

### Mitochondrial Reactive Oxygen Species (ROS)

The intensity of mitochondrial ROS was analyzed by fluorescent microscopy. C6 ceramide or C6 dihydroceramide treated HCSF (48 h post-plating) grown in 6 wells plates briefly washed with HBSS containing Ca^2+^ and Mg^2+^ and then incubated with DMSO soluble 5 µM MitoSOX^TM^ Red Superoxide Indicator(M36008, Invitrogen, Madison, WI) for 10 min at 34°C, protected from light. The cells were washed, counterstained with 5 µl/10 ml Hoechst 33342(Invitrogen) in HBSS for another 10 minutes and used for imaging.

### Mitochondrial Isolation

Mitochondria from HCSF were isolated using Pierce mitochondrial isolation kit (89874, Pierce/Thermo, Rockford, IL) as per option ‘A’ of the kit guidelines. Briefly, 2×10^7^HCSF grown in T150 flask were pre-incubated for 3 h, 6 h and 12 h with C6 ceramide or C6 dihydroceramide. Floaters along with attached cells were pooled and centrifuged together (1000×g/5 minutes). Cytosolic fraction was collected separately while mitochondrial pellet was resuspended in RIPA buffer (sc 24948, Santa Cruz Biotechnology, Santa Cruz, CA) mixed with Na_3_VO_4_, proteinase inhibitor cocktail, and PMSF. Protein concentrations were determined with the BCA Protein Assay Reagent Kit (Pierce/Thermo Scientific, Rockford, IL). Both cytosolic and mitochondrial fractions were stored at −80°C till their use for Western.

### Immunoblot Analysis

Media was removed and cells were lysed in modified RIPA lysis buffer(Santa Cruz Biotech) supplemented with PMSF, Na_3_VO_4_, and 1∶10 protease inhibitor cocktail in 1X PBS made from tablets (14654600, Roche Diagnostics, Indianapolis, IN). Lysates were prepared by freeze thawing, Dounce homogenization, and sonicated twice (15-s intervals at 100 W power). Cell debris was cleared from the lysates (centrifuged 200× g for 15 centrifuged min at 4°C) and protein concentrations were determined with the BCA Protein Assay Reagent Kit (Pierce/Thermo Scientific, Rockford, IL). The lysates were stored at -80°C till their use. For Western blotting 15 µg to 30 µg proteins were loaded in each lane of Tris HCl Criterion^ TM^ 10% or 18%Tris-HCl polyacrylamide gels(BIO-RAD, Hercules, CA). SDS-PAGE was run and proteins were electrophoretically transferred to PVDF filters. Filters were blocked with 5% serum or non fat milk (1 h) followed by overnight incubation at 4°C with primary antibodies in suitable dilutions. Proteins were detected using the corresponding horseradish peroxidase-conjugated antibody and Amersham ECL western blotting reagent (RPN 2209, GE Healthcare, Little Chalfont; UK), according to the manufacturer's directions. Development of the blots was done with Amersham Hyperfilm ECL^TM^ high performance CL Film (GE Healthcare, Little Chalfont; UK), using Kodak GBX developer and fixer solutions (Pierce/Thermo Scientific, Rockford, IL). Blots were re-probed after removing the antibodies and substrates with the Restore Western Blot Stripping Buffer (Pierce/Thermo, Rockford, IL), according to the manufacturer's directions. Protein loading was normalized by β-actin or β-tubulin signal,

### Co-IP Studies

Samples prepared from C6 ceramide or C6 dihydroceramide treated cells were lysed in IP lysis buffer. Pre-cleared lysates were subjected to IP for overnight at 4°C with 0.002 µg/µl anti-p32 or anti-BAD and additional 2 centrifuged hrs incubation at room temp with agarose G beads (20398, Pierce/Thermo, Rockford, IL). The IP mixtures were centrifuged at 8000×g for 5 minutes and the pellet was washed four times with (1X) washing buffer (23225, Pierce/Thermo, Rockford, IL). The complex was resuspended in RIPA buffer, and proteins were eluted with SDS buffer for Western analysis to detect protein interactions among BAD, HRK and P32.

### Small interfering RNA treatment of cells

SiRNA complexes were formed in serum-free medium, using INTERFERIN (PolyPlus; Genesee Scientific, San Diego, CA), according to the manufacturer's instructions. Optimal siRNA/INTERFERIN concentrations were established. Cells were plated at 45–50% confluence in DMEM containing 5% FBS, Mito+serum extender (Gibco, Carlsbad, CA), and ciprofloxacin and maintained at 34°C. 24 h post-plating, the media was removed and the cells rinsed with HBSS. The cells were treated with a final concentration of 40 nM siRNA. Cells were transfected with siRNA complexes in media composed of (1∶50) INTERFERIN: DMEM containing 5% FBS Mito+ and ciprofloxacin. After overnight incubation (16 h), the transfection media was replaced with regular culture media. 72 h post-transfection, cells were treated with 40 µg C6 ceramide or C6 dihydroceramide (6–16 h incubations) for relevant experiments.

### RNA isolation, cDNA synthesis, Real-time (q) PCR

Total RNA was prepared from cells using Trizol (Invitrogen) and was followed by DNA digestion with DNase I (Invitrogen, Madison, WI) and further purified by RNeasy minikit protocol (Qiagen, Valencia, CA). RNA concentrations were determined by measuring the optical density at 260, 280 and 320 nm. Equal amounts of RNA from each sample were then reverse transcribed to cDNA by using a Superscript first-strand synthesis kit (Invitrogen). qPCR were performed in an ABI 7500 (Applied Biosystems), using SYBR Green PCR Master Mix (Applied Biosystems) and 50–75 ng cDNA at 50°C for 2 min, 95°C for 10 min, 40 cycles of 95°C for 15 s, annealing for 1 min at 60°C, then extension at 72 °C for 45s. HRK specific primers (PPH00369A, SA Biosciences, Frederick, MD) and glyceraldehyde-3-phosphate dehydrogenase (GAPDH) (S) *5′- ctctctgctcctcctgttcg-3′*, (AS) *5′-tgactccgaccttcaccttc-3*′ primers were used in the concentrations of 10 pM/20 µl. Melt curve analysis was performed by an additional dissociation step of 1 cycle at 95°C for 15 s and ramping data collection at 60°C for 1 min and 95°C for 15 s. C_t_ values were calculated using ABI software. Data were normalized against the GAPDH signal. Relative expression values were obtained by normalizing C_t_ values of the tested genes with C_t_ values of the housekeeping genes, using the ΔΔC_t_ method.

### Statistical Analysis

Means were compared by t test or one-way analysis of variance, and the significance of differences among means of treatment groups was determined using Sigma Stat software (Sigma Stat 3.0; SPSS Inc., Chicago, IL).

## Results

### Ceramide Caused HCSF Death

**Figure 1:**
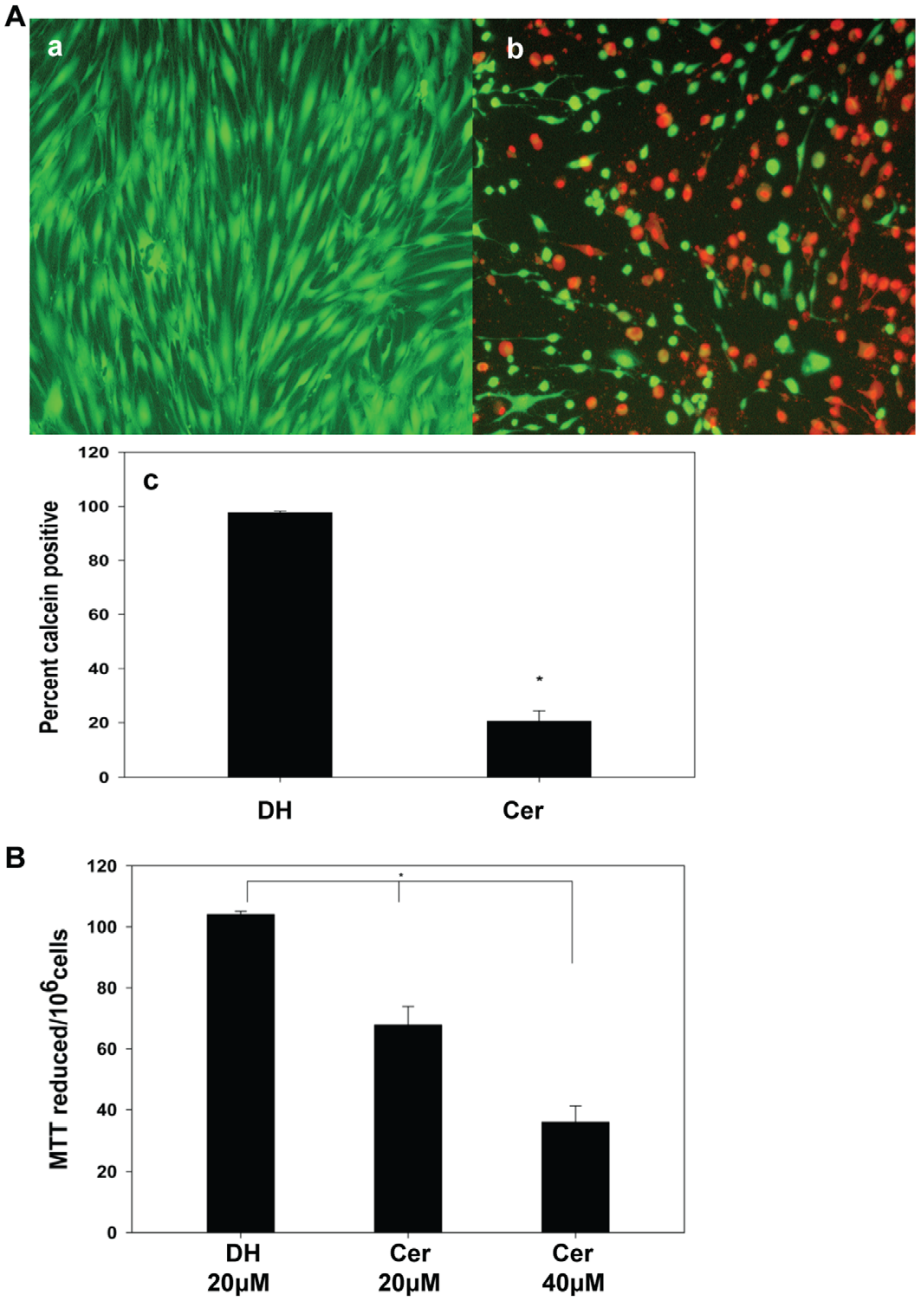
Ceramide induced cell death. **A:** HCSF were grown to confluence in DMEM (without phenol red) containing 5%FBS, Mito+, and ciprofloxacin. The cells were treated with (a) 40 µM C6 dihydroceramide or (b) 40 µM C6 ceramide for 16 hrs, and then briefly washed with HBSS containing Ca^2+^ and Mg^2+^ and incubated with calcein AM and ethidium nuclear stain (Invitrogen) for 30 min at 37°C. Cell death was determined by fluorescent microscopy. Cell counts of dead cells were expressed as percentage of total cells counted. (c) The percent of calcein AM positive cells (Mean±SEM) was significantly reduced in the C6 ceramide treated culture relative to C6 dihydroceramide treated controls (Mean±SEM) (P = 0.008 Mann-Whitney Rank Sum test). **B:** Assessment of cell death was also determined by MTT assay following treatment with C6 ceramide. HCSF were grown to confluence in DMEM (without phenol red) containing 5% FBS, Mito+, and ciprofloxacin. The cells were treated for 16 hrs with (1) 40 µM C6 dihydroceramide, (2) 20 µM C6 ceramide or (3) 40 µM C6 ceramide. Ceramide induced cell death was measured by formazan formation. MTT (3-(4, 5-Dimethylthiazol-2-yl)-2, 5- diphenyltetrazolium bromide, a tetrazole) was reduced to formazan by living cells. C6 ceramide treatment decreased the formazan formation (Mean±SEM) compared to C6 dihydroceramide treated controls (Mean±SEM). (*)Statistically significant (p<0.001) as determined by ANOVA with multiple comparisons (Holm-Sidak method).

We observed that exogenous treatment with short chain ceramides (C-2 or C-6) caused dose dependent (0 to 80 µM) death of HCSF in manner similar to that observed with other cell types [22]. For example, C6 ceramide significantly reduced the cell viability compared to dihydroceramide or no treatment within 16 hours of exposure to HCSF (Figure 1). In the studies reported here cell viability was quantified using calcein AM/EthD-1 double staining (Figure 1A). Using the MTT assay, an indicator of mitochondrial activity, we confirmed that ceramide treatment of HCSF caused mitochondrial dysfunction, a likely cause of the loss of cell viability (Figure 1B) [23], [24].

### Ceramide Induced HCSF Death and Mitochondrial Dysfunction

**Figure 2:**
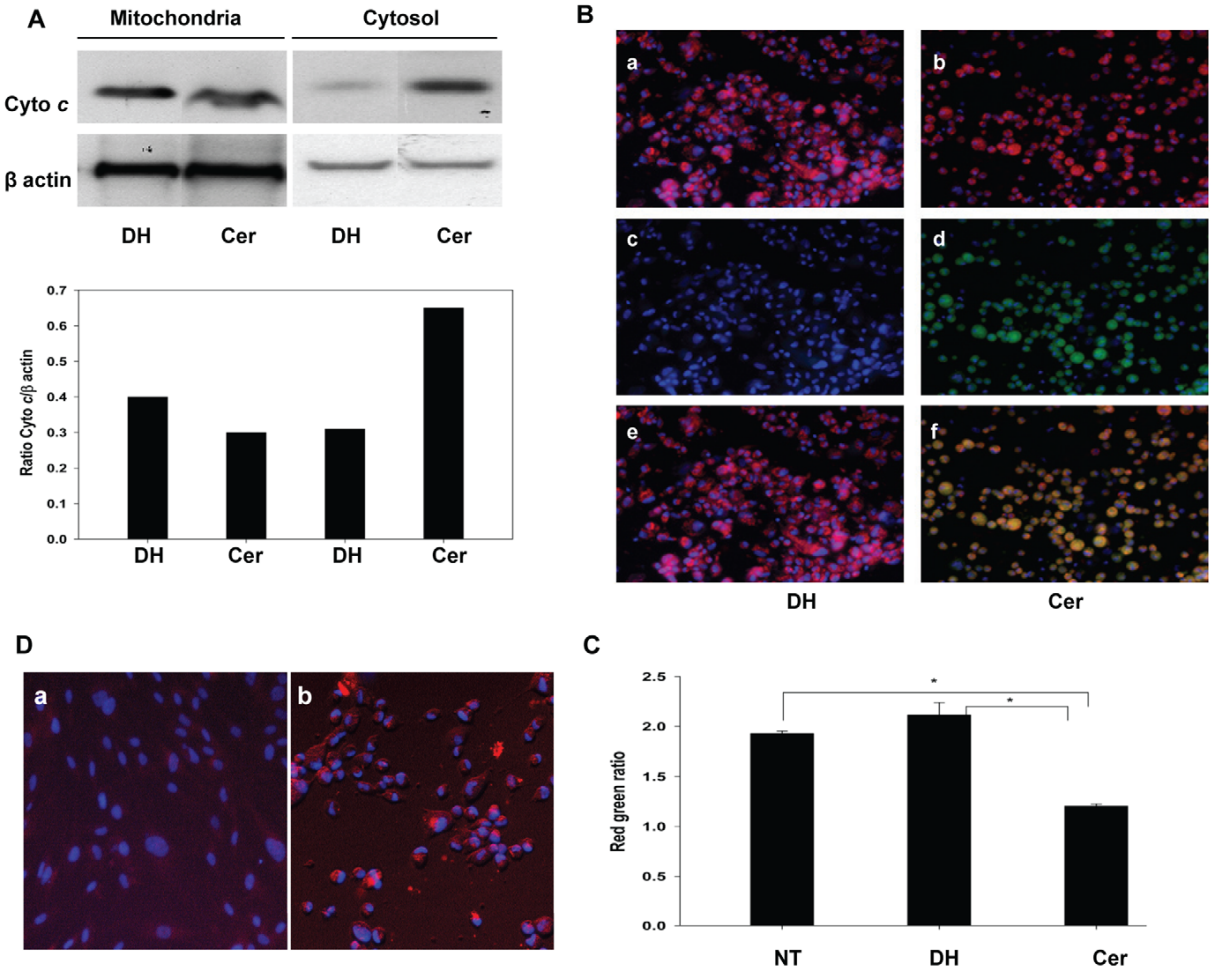
Ceramide treatment caused HCSF mitochondrial dysfunction. **A:** C6 ceramide induced cytochrome c (cyto *c*) release from mitochondria to cytoplasm was determined by Western blotting. HCSF grown in DMEM containing 5%FBS, Mito+, and ciprofloxacin were treated with 40 µM C6 dihydroceramide or C6 ceramide for 16 hrs. Western blot of cell extracts from mitochondrial and cytosol fraction using antibodies to cyto c showed release of cyto c from mitochondria. Vertical bars represent ratio of cyto c to β-actin (arbitrary values of relative quantification normalized to loading control β-actin) (1) C6 dihydroceramide treated or (2) C6 ceramide treated mitochondrial fractions; (3) C6 dihydroceramide treated or (4) C6 ceramide treated cytosol fractions. **B:** Mitochondrial membrane potential (Δψm) changes were determined by fluorescence microscopy. In cells treated with JC-1 the mitochondria of healthy cells fluoresce red while cells undergoing death fluoresce green when the mitochondrial potential collapses. (b) C6 ceramide treated cultures stained with JC-1 show less intense J aggregate compared to C6 dihydroceramide treated control (a); (d) green fluorescence indicating Δψm loss compared to healthy mitochondria(c); (e) merged images (a and c), (f) merged images (b and d). **C:** Mitochondrial membrane potential (Δψ_m_) red/green ratios were measured by fluorescence emission of red and green fluorescence using a fluorescence plate reader. (NT) No treatment, (DH) C6 dihydroceramide control, (Cer) C6 ceramide treated cultures. C6 ceramide treatment decreased the red green ratio (Mean±SEM) compared to controls (Mean±SEM) indicating Δψ_m_ loss. (*) Statistically significantly different from no treatment or DH treated groups. (p<0.05) ANOVA with multiple comparisons (Holm-Sidak method) was performed. **D:** Mitochondrial reactive oxygen species (ROS) was analyzed by fluorescence microscopy. Cells grown in DMEM containing 5% FBS (without phenol red) were treated with C6 ceramide or C6 dihydroceramide for 16 hours. Cells were washed with HBSS containing Ca^2+^ and Mg^2+^ and incubated with MitoSOX Red for 10 min at 37°C, protected from light. The cells were washed and counterstained with Hoechst 33342. MitoSOX Red-emitted fluorescence intensified in C6 ceramide treated cultures indicating an increased in ROS levels. (a) C6 dihydroceramide treated cells, (b) C6 ceramide treated cells.

Release of cyto *c* from the mitochondria into the cytosol has been observed to be among the ceramide regulated mitochondrial properties that influence cell survival [25], [26], [27]. Our data document cyto *c* immunoreactivity in the cytosolic fraction prepared from C6 ceramide treated HCSF undergoing cell death. In contrast cyto *c* was confined to mitochondria in C6 dihydroceramide treated control cells (Figure 2A). To evaluate mitochondrial ROS production and membrane potential we employed MitoSOX Red and JC-1 fluorescent probes, respectively. Our data revealed that C6 ceramide exposure significantly enhanced mitochondrial ROS production as evident from the increased intensity of red fluorescence in C6 ceramide treated HCSF (Figure 2B). Furthermore we observed a fall in red to green fluorescence ratio in the HCSF treated with ceramide and exposed to JC-1 (Figure 2C, 2D). The mitochondrial Δψ_m_ clearly decreased in C6 ceramide treated HCSF compared to C6 dihydroceramide treated counterparts. Thus ceramide treated HCSF released cyto *c* into the cytosol, increased the production of ROS and possessed a compromised the Δψ_m._ All of these alterations in mitochondrial functions are believed to contribute to cell death [2], [10].

### Ceramide Induced HRK Expression linked to Mitochondrial Dysfunction and Cell Death

**Figure 3:**
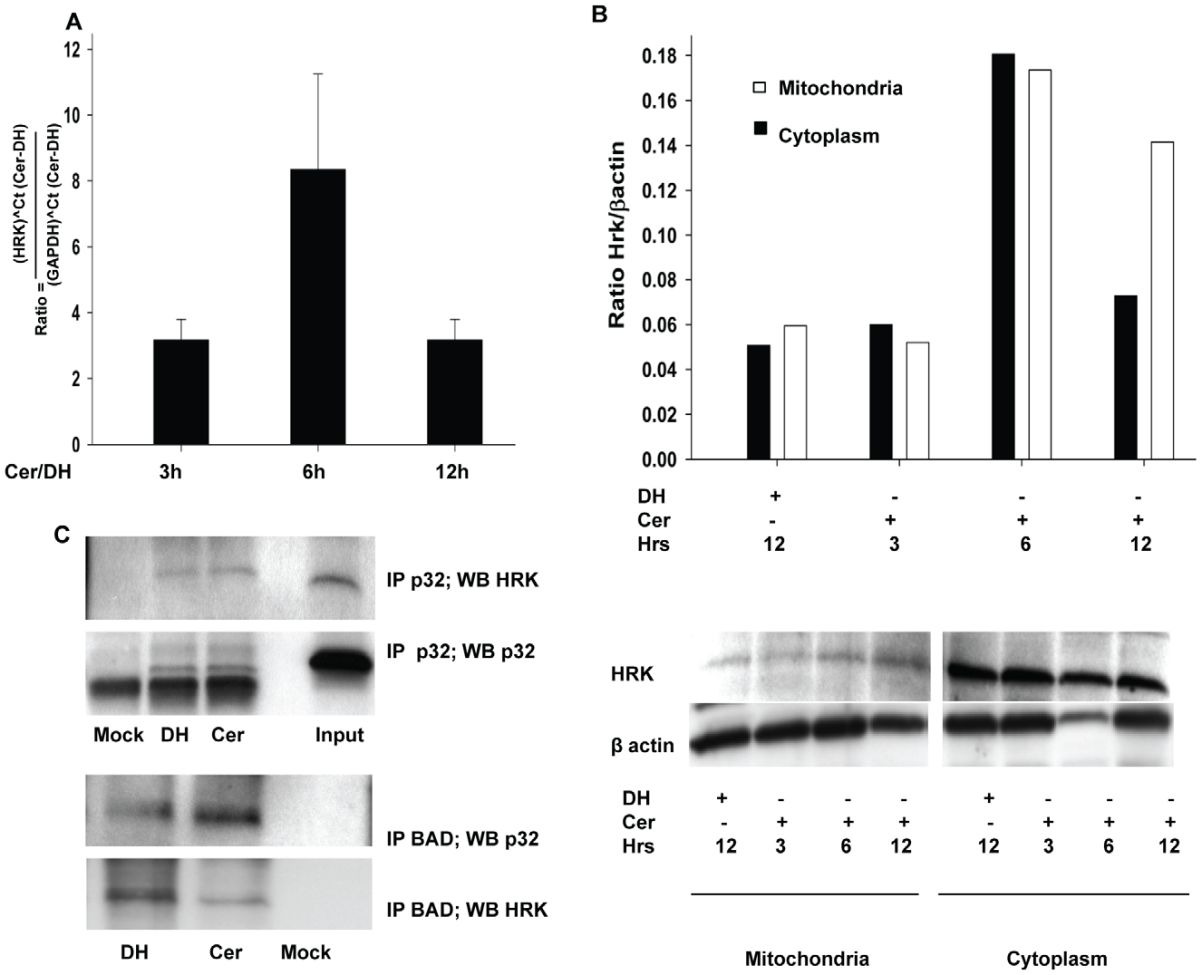
Interaction of HRK with Bad and p32. **A:** Ceramide induced HRK expression. HCSF were grown to confluence in DMEM containing 5%FBS, Mito+, and ciprofloxacin. The cells were treated with 40 µM C6 ceramide for 3, 6 or 12 hours and compared with 40 µM C6 dihydroceramide as control group. Real time (q) PCR using Hrk specific primers documented increased steady state pools of HRK mRNA at 6 hrs by C6 ceramide treatment compared to C6 dihydroceramide. Vertical bars represent fold change (Mean ± SEM) by C6 ceramide treatments with respect to dihydroceramide treatments at (1) 3 hours, (2) 6 hours and (3) 12 hours. **B:** Ceramide induced HRK translocation to mitochondria. HCSF grown in DMEM containing 5%FBS, Mito+, and ciprofloxacin were treated with 40 µM C6 ceramide for 3, 6 and 12 hours. (a)Western blot of cell extracts from mitochondrial and cytosol fraction using antibodies to HRK was performed. Vertical solid and hollow bars respectively represent ratio of HRK to β-actin in cytoplasm and mitochondria. (1) C6 dihydroceramide treated control cultures or C6 ceramide treated cultures (2) 3 hrs, (3) 6 hrs (4) 12 hrs. **C:** Protein interaction among mitochondrial p32, HRK and BAD. Cell lysates were prepared from 40 µM C6 ceramide and C6 dihydroceramide treated HCSF and Co-immunoprecipitations were performed. The lysate was pre-cleared by G agarose and subjected to IP for overnight at 4°C with either anti-BAD or anti-p32 antibodies. HRK interaction with p32 and Immunoblot (WB) with anti-HRK), HRK interaction with BAD (IP with BAD; WB with anti-HRK), and p32 interaction with BAD (IP with BAD; WB with anti-p32) was determined by Western analysis. Non-immune host serum source of anti-p32 and anti BAD was used as mock control.

Preliminary studies using PCR arrays to assess the expression of genes related to apoptosis following the first 6 to 12 hr post ceramide treatment of HCSF indicated that the HRK gene was significantly up regulated (data not shown). Using HRK specific primers for qPCR and HRK specific antibodies for western analysis we confirmed the preliminary observation made using the arrays. *HRK* gene expression peaked in ceramide treated cells 6 h post C6 ceramide treatment compared to C6 dihydroceramide or no treatment control (Figure 3A). Western analysis documented increased in HRK protein between 6 to 12 hours post-C6 ceramide treatment. HRK protein became associated with mitochondria in samples from C6 ceramide treated HCSF in 12 hours (3B). HRK (Harakiri) belongs to the BH3 only protein family originally identified in rat sympathetic neurons [28] and in HeLA cells [29]. *HRK* expression has been demonstrated to play a role in initiating cell death under physiological and pathological conditions [19], [30]. HRK has been detected in tissues including but not limited to brain, lymphoid tissues, pancreas, liver, lung, and kidney [31]. This is the first report if its detection in tissues of the eye. In oligodendrocytes it appeared that HRK was associated with death by apoptosis [18]. Based on this information, we envisaged ceramide induced *HRK* expression in HCSF could be involved in cell death mediated by mitochondrial dysfunction but as described below the process of ceramide induced cell death of HCSF appeared to be more complicated that a pure apoptotic process.

### HRK-P32-BAD Interactions are Involved in Ceramide Induced Mitochondria Dysfunction

Western blotting showed the HRK protein was primarily localized in mitochondria (Figure 3B). Yeast two hybrid studies have shown the existence of HRK interaction with mitochondrial p32 [19] in the cells undergoing apoptosis. We confirmed this association with mitochondrial p32 by applying co-immunoprecipitation. When p32 was immunoprecipitated, HRK was clearly associated with the p32 precipitated (Figure 3C). Furthermore, we demonstrated that just like HRK, co-immunoprecipitation of BAD with p32 revealed that in HCSF, BAD too was associated with p32 (Figure 3C) under the influence of ceramide [12]. Thus in ceramide treated HCSF interactions among HRK, p32 and BAD in the mitochondria were likely contributor to cell death (Figure 3C).

### Ceramide Caused JNK Phosphorylation is Involved in *HRK* Expression

**Figure 4:**
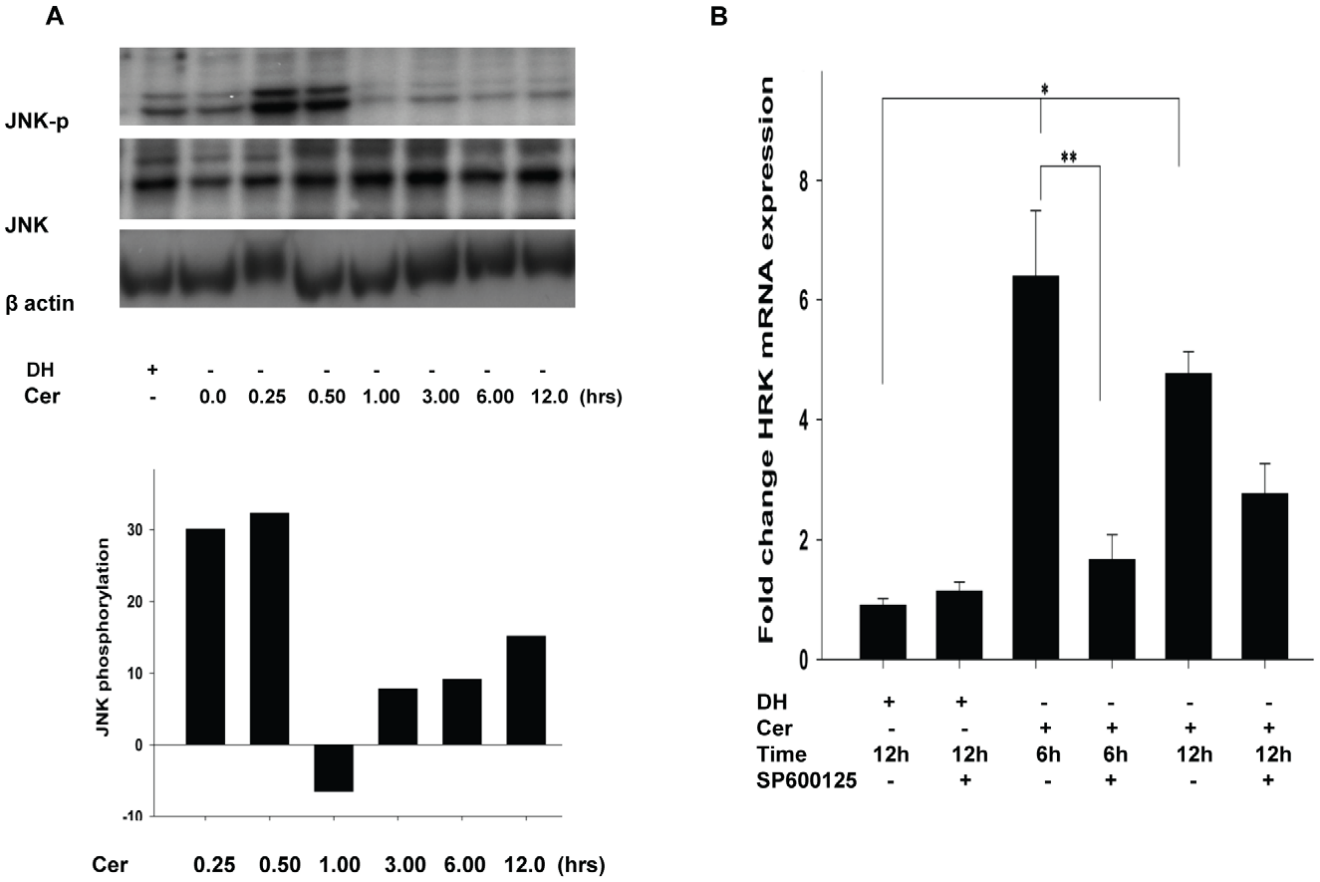
Ceramide induced JNK phosphorylation regulates HRK expression. **A:** HCSF grown in DMEM containing 5%FBS, Mito+, and ciprofloxacin were treated with 40 µM C6 dihydroceramide as control or 40 µM C6 ceramide at various times. Western blot of cell lysates using antibodies to (a) phospho-JNK (JNK-p), (b) JNK and (c) β actin demonstrated peak phosphorylation of JNK 30 to 60 minutes post-treatment. **B:** Vertical bars represented as percent phosphorylation caused by ceramide with respect to C6 dihydroceramide. The percentage phosphorylation was calculated as difference in the arbitrary values between ceramide and dihydroceramide obtained as ratio by relative quantifications. The ratios were calculated and normalized to β actin. **C:** JNK regulates HRK expression. HCSF grown in DMEM containing 5%FBS, Mito+, and ciprofloxacin were treated with 40 µM C6 dihydroceramide (DH) as control or 40 µM C6 ceramide at various times with, or without 10 µM JNK inhibitor SP600125. qPCR was performed.(1) 40 µM DH, (2) 40 µM DH+SP600125, (3) 40 µM C6-ceramide(6 hrs), (4) 40 µM C6-ceramide (6 hrs)+ SP600125, (5) 40 µM C6-ceramide (12 hrs), (6) 40 µM C6-ceramide (12 hrs) + SP600125. Data were analyzed using ΔΔCt method and normalized against GAPDH as housekeeping gene relative to no treatment control (mean±SEM). Statistically significant ((*) p<0.001), ((**) p<0.05) ANOVA with multiple comparisons (Holm-Sidak method).

Ceramide has been shown to activate stress-related kinases, including stress-activated protein kinases (SAPKs) [32] and c-jun NH2 terminal kinase (JNK) [33]. Western blots using anti JNK-p to probe lysates prepared from C6 ceramide or C6 dihydroceramide treated HCSF demonstrated increased JNK phosphorylation in the HCSF samples prepared 0.25 to 0.50 h post-ceramide treatment relative to C6 dihydroceramide treated samples (Figure 4A). JNK has been implicated in *HRK* expression [30]. In our studies C6 ceramide induced *HRK* expression was significantly ablated by more than 75 percent in 6 hour and about 30 percent in 12 hours when the JNK inhibitor, SP600125, was added to the cell culture media (Figure 4B).

### HRK Knockdown of Ceramide Induced Death and Mitochondrial Dysfunction

**Figure 5:**
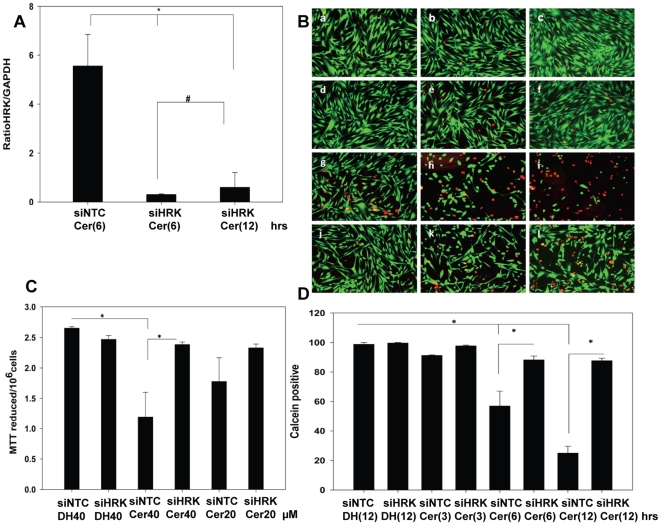
siHRK knockdown HRK mRNA and ceramide induced cell death. **A:** HRK knockdown of steady state pool of HRK mRNA. HCSF grown in DMEM + 5% FBS were transfected with 40 nM siHRK or 40 nM siNTC (as control) for 72 hours. Cells were then treated with 40 µM (DH) control or 40 µM C6 ceramide for 6 to 12 hours. qPCR was performed (1) siNTC(control)+ 40 µM C6 ceramide (6 h), (2) siHRK+ 40 µM C6 ceramide (6 h), (3) siHRK + 40 µM C6 ceramide (12 h). Data were analyzed using ΔΔCt method and normalized against GAPDH as housekeeping gene. The fold change in HRK expression was calculated with respect to siNTC+DH (12 h) treated control. (*) Statistically significant, (#) statistically not significantly different, (p<0.05) ANOVA with multiple comparisons (Holm-Sidak method) was performed. **B:** HRK knockdown ceramide induced cell death. HCSF were grown HCSF grown in DMEM +5% FBS were transfected with 40 nM siHRK or 40 nM siNTC for 72 hours. Cells were then treated with 40 µM DH and 40 µM C6 ceramide for 3, 6 and 12 hours. Cells washed briefly with HBSS containing Ca^2+^ and Mg^2+^ and then incubated with calcein AM and ethidium nuclear stain for 30 min at 37°C. Cell death was determined by fluorescent microscopy(a) siNTC +DH(3 h),(b) siNTC +DH(6 h),(c) siNTC +DH(12 h),(d) siHRK +DH(3 h), (e) siHRK +DH(6 h), (f) siHRK +DH(12 h),(g) siNTC + ceramide(3 h),(h)siNTC + ceramide(6 h),(i) siNTC + ceramide(12 h) (j) siHRK + ceramide(3 h),(k) siHRK + ceramide(6 h), (l) siHRK + ceramide(12 h). **C:** Assessment of siHRK transfected cell survival by MTT assay following treatment with C6 ceramide. HCSF grown in DMEM +5% FBS were transfected with 40 nM siHRK or 40 nM siNTC (as control) for 72 hours. Cells were then treated with the following for next 12 hours (1) siNTC+ 40 µM C6 dihydroceramide, (2) siHRK+ 40 µM C6 dihydroceramide or (3) siNTC+ 40 µM C6 ceramide, (4) siHRK+ 40 µM C6 ceramide or (5) siNTC+ 20 µM C6 ceramide, (6) siHRK+ 20 µM C6 ceramide. Cell survival by siHRK was assessed by formazan formation. MTT (3-(4, 5-Dimethylthiazol-2-yl)-2, 5- diphenyltetrazolium bromide, a tetrazole) was reduced to formazan by living cells. (*) Statistically significant (p<0.05) ANOVA with multiple comparisons (Holm-Sidak method). **D:** HRK knockdown prevents ceramide induced cell death. Panel showing cell survival by siHRK following C6 ceramide treatment, assayed by calcein AM and ethidium nuclear stain. Vertical bars represent percentage of calcein positive cells relative to total cells counted. (1) siNTC+ 40 µM +C6 dihydroceramide (12 hrs), (2) siHRK+ 40 µM C6 dihydroceramide (12 hrs), (3) siNTC+ 40 µM C6 ceramide (3 hrs), (4) siHRK+ 40 µM C6 ceramide(3 hrs), (5) siNTC+ 40 µM C6 ceramide (6 hrs), (6) siHRK+ 40 µM C6 ceramide (6 hrs), (7) siNTC+ 40 µM C6 ceramide(12 hrs), (8) siHRK+ 40 µM C6 ceramide (12 hrs). (*) Statistically significant (p<0.05) ANOVA with multiple comparisons (Holm-Sidak method) was performed.

HCSF were transfected with 40 nM siHRK or siNTC (non target control siRNA) and were subjected to C6 ceramide or C6 dihydroceramide treatment. We found HRK siRNA treatment significantly reduced steady state mRNA pools of HRK in HCSF treated with C6 ceramide for 6 or 12 hours compared to siNTC transfected cells that had been treated with ceramide (Figure 5A). Cell viability of HCSFs that were transfected with either siHRK or siNTC and treated with C6 ceramide or C6 dihydroceramide for 3 h, 6 h or 12 h was assessed by calcein AM/Eth-D1 double staining. We observed siHRK transfection of HCSFs more effectively rescued cell survival(Figure 5B-j,k,l) compared to HCSFs transfected with siNTC and treated with C6 ceramide for 3 h,6 h and 12 h (Figure 5B-g,h,i). Thus transfection with siHRK significantly knocked-down the ceramide induced cell death (Figure 5C). The MTT assay was used to verify the pro-survival role of siHRK. We observed cells transfected with siHRK were able to reduced MTT to formazan more efficiently than HCSF transfected with siNTC when treated with C6 ceramide (Figure 5D).

## Discussion

In this study we demonstrated: (1) mitochondrial dysfunction and cell death in C6 ceramide treated HCSF by the MTT assay indicating that mitochondrial electron transport had been disrupted; cell death was confirmed using the calcein AM assay; (2) the release of cyto *c* from mitochondrial inter membrane space (IMS); (3) the loss of ΔΨ_m_ loss and (4) enhanced production of mitochondrial ROS. C6 ceramide induced HRK expression was mediated by JNK. HRK was translocated to mitochondria from the cytosol where it interacted with BAD and mitochondrial p32. Ceramide induced cell death was significantly reduced by HRK siRNA treatment. Our data provide the first evidence that HCSF death following ceramide exposure is due to mitochondrial dysfunction mediated by expression of HRK. Mitochondrial dysfunction is the critical step involved in C6 ceramide activated HRK mediated demise of HCSF.

The loss of mitochondrial integrity and function leads to pathological conditions related to several diseases such as: diabetes, cardiovascular disorders, neurodegenerative disorders, and diseases of eye [14], [34]. Recently, mitochondrial dysfunction has been implicated in corneal cell death related to dry eye disease and keratoconus which contribute to loss of corneal transparency and decreased vision acuity [13], [14]. The hallmarks of mitochondrial dysfunction include generation of reactive oxygen species, ATP depletion, collapse in the inner mitochondrial membrane potential, inhibition and/or activation of the mitochondrial electron transport chain, and release of IMS proteins [11]. In this study, up-regulated expression of HRK (Figure 3A), release of cyto *c* in the cytosol (Figure 2D), decreased percent of calcein-AM positive cells (Figure IA) and decreased reduction of MTT (Figure 1B) characterize HCSF cell death mediate by ceramide. Depending on the cell type, death stimulus, and experimental conditions, multiple molecular mechanisms of mitochondrial dysfunction may co-exist involving the cyto *c* release, permeability transition pore (PTP) complex activity, and the BAX/BAK pore-forming properties [26], [27], [35] in causing the MOMP. Regardless of the mechanisms of cyto *c* release and ΔΨ_m_ loss, once MOMP occurred, cells appear to reach the “point of no return” and their fate appears to be sealed [36], [37]. Our experimental model illustrates the basic tenets of a mitochondrial death pathway as manifested by the defective function of complexes II and III of respiratory chain in the form of decreased MTT reduced (Figure 1B); increased localization of cyto *c* in the cytosol (Figure 2D); increased production of mitochondrial ROS (Figure 2A) and loss of ΔΨ_m_ (Figure 2B and 2C).

Ceramides regulate BH3 only proteins such as BID [38], BAD [16] and HRK [18] directly and thus affect complex III of respiratory chain [38] resulting in release of cyto *c* into the cytosol [27]. In our study we found C6 ceramide caused JNK phosphorylation in regulating HRK production (Figure 4A and 4B). C6 ceramide activated HRK transcription was likely due to the presence of conserved ATF binding site in *HRK* promoter region, where c Jun binds after phosphorylation [39]. Ceramide treatment not only induced HRK expression at mRNA and protein levels but also enhanced its translocation to mitochondria by 12 hours (Figure 3A, 3B) for the release of *cyto c*. BH3 proteins undergo posttranslational modification (PTM) in causing inner mitochondrial membrane remodeling for cyto *c* release from cristae [4], [12], [27], [39]. We have not study the absence or presence of PTM in HRK, nonetheless; we have observed HRK interaction with mitochondrial p32 (Figure 3C), which is a critical regulator of mitochondrial membrane potential and cell death [20]. The interaction of HRK with p32 was necessary for HRK to exert its pro-death activity [19]. In these studies we have also observed that HRK siRNA mediated knockdown of HRK resulted in a decrease in steady state pool of HRK mRNA and reduced the ability of ceramide to cause mitochondrial dysfunction and cell death (Figure 5A–D). Thus, it appears that HRK/p32 interactions are capable of activating a mitochondrial death pathway in HCSF.

P32 may be involved in regulating the mitochondrial concentrations of Ca^2+^ and permeability transitions of the inner membrane in association with the PTP complex for releasing the cyto *c* [40]. We performed *Insilco analyses* that revealed the homology existing among p32, HRK and BAD in the regions of pre-BH3, BH3 and post-BH3 domains. Homologies also exist in the N terminal region (aa1-73) of p32 which contains the signal sequence of p32 that targets mitochondria (Figures S1 and S2) [19]. In view of the interactions observed among BAD,mitochondrial p32 and HRK (Figure 3C) it has been hypothesized that these complexes are likely to be involved in signaling associated with the ceramide induced regulation of Ca^2+^ and PTP sensitization in contributing to the mitochondrial dysfunction and eventually to HCSF death(Figure 2B and 2C). Thus, ceramide may act as a stimulus of death that causes PTMs in BH3 proteins by creating active conformations responsible for mitochondrial dysfunction resulting in the loss of ΔΨ_m_ and cyto *c* release to the cytosol due to PTP opening of the inner mitochondrial membrane [4].

We have observed increased production of mitochondrial ROS (Figure 2A); however, we are not sure about the reason for increased ROS production following ceramide challenge. The reduction in ΔΨ_m_ coupled with increased generation of ROS may be due to the induction of NADPH oxidase (NOX) expression [25], [41]. We observed that superoxide can be produced in HCSF by NADPH oxidases mediated by complexes containing NOXs 1, 4, and 5 [21]. We also have observed the induction of NOX by ceramide treatment (Rizvi et al unpublished). Therefore we suggest that ceramide induced NOX may contribute to the mitochondrial death signaling.

Clearly; our findings provide first evidence that BH3-only proteins, such as HRK and BAD maybe capable of directly interacting with mitochondrial protein p32 in HCSF. In this way they are more close to hierarchy model of BH3-only proteins activation involving mitochondrial mediated death of HCSF following the stimulus by ceramide [6]. These protein interactions along with the generation of ROS and JNK signaling appeared to be the key events in the cells response to ceramide and may help in defining the mechanism of ceramide mediated death of HCSF in the present paradigm. These observations further suggest the possibility of endoplasmic reticulum stress and mitochondrial dysfunction (ERSMD) contributing to the demise of ceramide treated HCSF [11], [12]. As ER stress and JNK signaling pathway have been linked with both HRK activation and ROS generation by NADPH oxidases and thereby linked to the process of mitochondrial permeabilization and cell death [29], [42], [43]. Due to the fact that although annexin v staining, activation of caspase 3 on ceramide treatment (data not shown) and TUNEL staining occur in a portion of the population and not to the extent expected in classic apoptosis it is unclear whether to suggest that the cells were dying due to necrosis, apoptosis, or the combination of both (Figures S3 and S4). Additional studies will be required to investigate the specific nature of ceramide induced cell death, the upstream events causal for JNK phosphorylation and the downstream events followed by HRK translocation to mitochondria in order to delineate the precise signaling mechanism operative in the mitochondrial dysfunction for the demise of HCSF. Better comprehension of such mechanism(s) may lead to the identification of new targets for drugs used to regulate corneal wound healing and maintenance of corneal clarity.

## Supporting Information

Figure S1 **Figure showing amino acid sequence alignment between HRK and p32 in the region of Pre BH3, BH3 and post BH3 domains.** Alignment can also be seen with N terminal region of p32, amino acids 1–73 (Blue solid) which contains the signal sequence of p32 that target mitochondria. Sequence alignment was done using T-Coffee (see File S1).
(TIF)

Figure S2 **Figure showing amino acid sequence alignment between BAD and p32 in the region of Pre BH3, BH3 and post BH3 domains.** Alignment can also be seen with N terminal region of p32, amino acids 1-73 (Blue solid) which contains the signal sequence of p32 that target mitochondria. Sequence alignment was done using T-Coffee (see File S1).
(TIF)

Figure S3 **TUNEL positive cells present in cultures following C6-ceramide treatment. Cells were grown to confluence and treated overnight with 40 µM C6 ceramide (left panel), or 5 nM staurosporine (right panel).** Cells were harvested and stained using TUNEL kits. The cells were analyzed by flowcytometry. Alexa Flour 488-A stained represent BrdU positive cells and PE-A stained represent propidium iodide positive cells (see File S2).
(TIF)

Figure S4 **Annexin V-affinity, resulting from phosphatidylserine (PS) exposure at the outer leaflet of the plasma membrane, apoptotic cells can be distinguished from annexin V-negative living cells, by using fluorescent microscopy procedure.** When combined with propidium iodide (PI) the double labeling procedure allows a further distinction of necrotic (pink arrow head, annexin V-/PI+), early apoptotic (green arrow head, annexin V+/PI-) or late apoptotic/necrotic (yellow arrow head, annexin V+/PI+) cells. The cells were used for fluorescent microscopy; the images were captured and measured for green and red fluorescence. Cells were treated with dihydroceramide (a) or 40 µM C6 ceramide (b) (see File S2).
(TIF)

File S1 Reference for Figures S1 and S2.
(DOC)

File S2 Material and Methods for Figures S3 and S4.
(DOC)
