# Supplementary figures and images for: Mitochondrial Dysfunction Links Ceramide Activated HRK Expression and Cell Death
Source: PLoS One. 2011 Mar 31;6(3):e18137. doi: 10.1371/journal.pone.0018137 (PMC3069046; doi:10.1371/journal.pone.0018137)

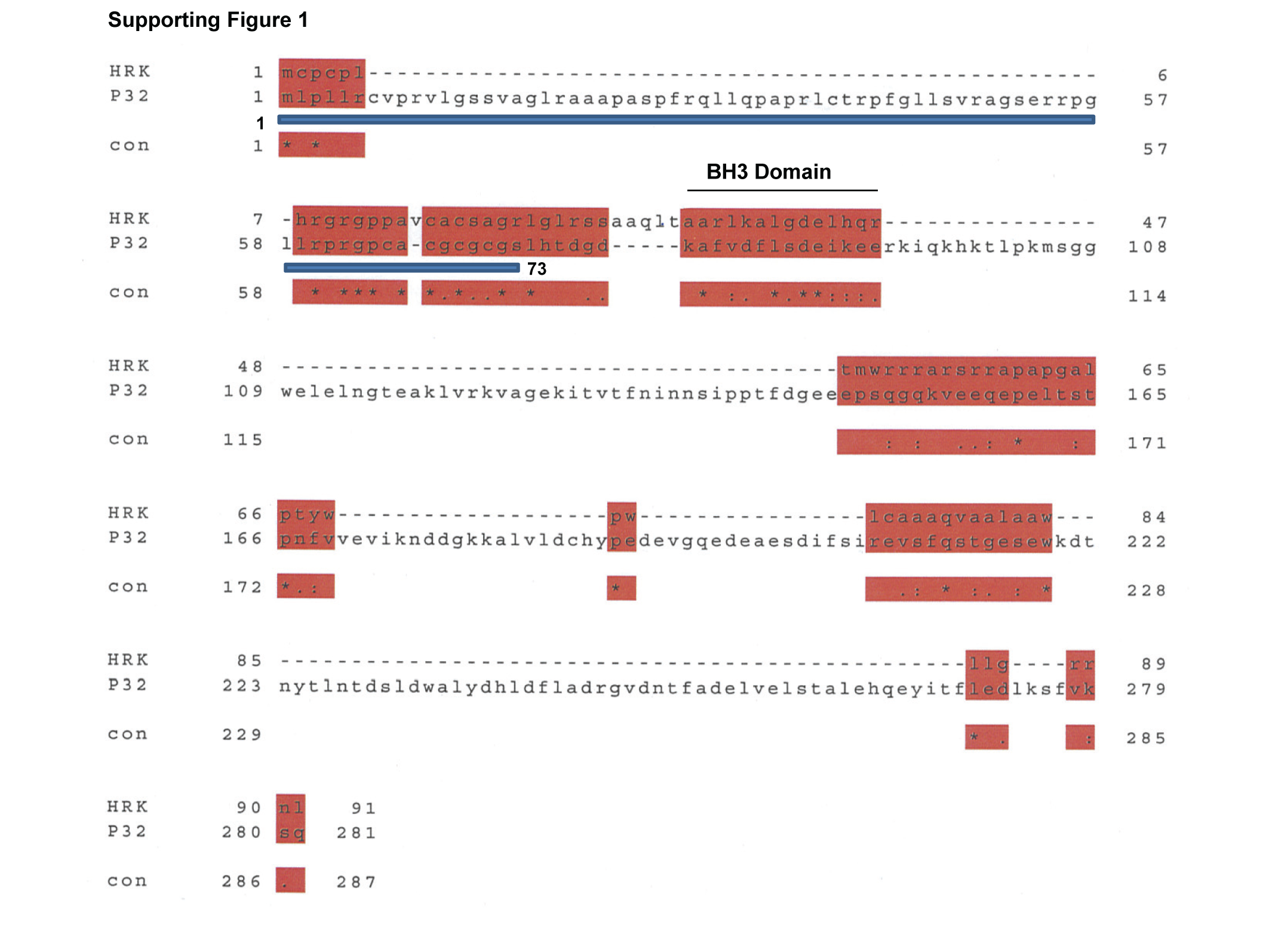

Supplement: Figure S1 — Figure showing amino acid sequence alignment between HRK and p32 in the region of Pre BH3, BH3 and post BH3 domains. Alignment can also be seen with N terminal region of p32, amino acids 1–73 (Blue solid) which contains the signal sequence of p32 that target mitochondria. Sequence alignment was done using T-Coffee (see File S1). (TIF) [file pone.0018137.s001.tif]

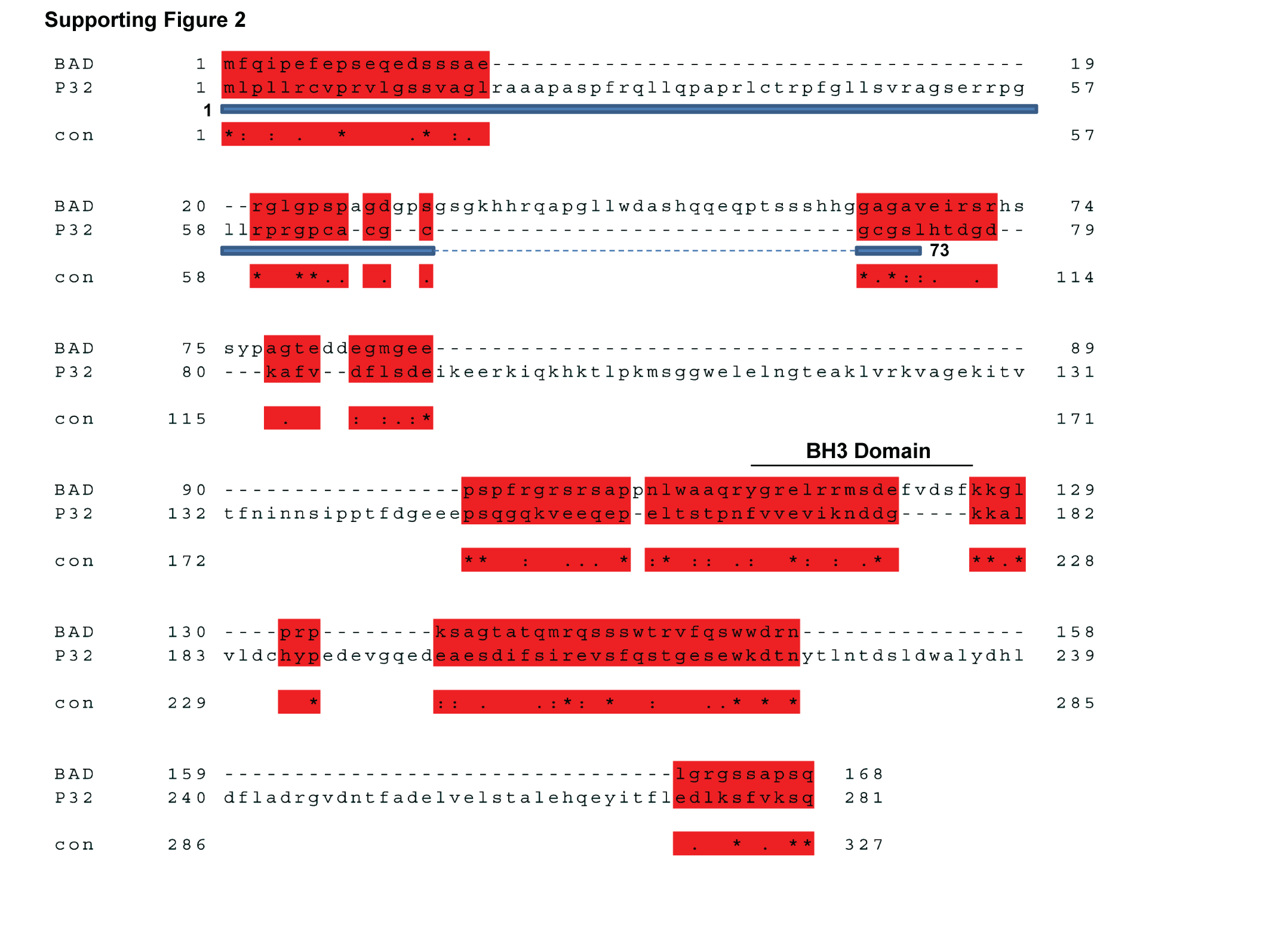

Supplement: Figure S2 — Figure showing amino acid sequence alignment between BAD and p32 in the region of Pre BH3, BH3 and post BH3 domains. Alignment can also be seen with N terminal region of p32, amino acids 1-73 (Blue solid) which contains the signal sequence of p32 that target mitochondria. Sequence alignment was done using T-Coffee (see File S1). (TIF) [file pone.0018137.s002.tif]

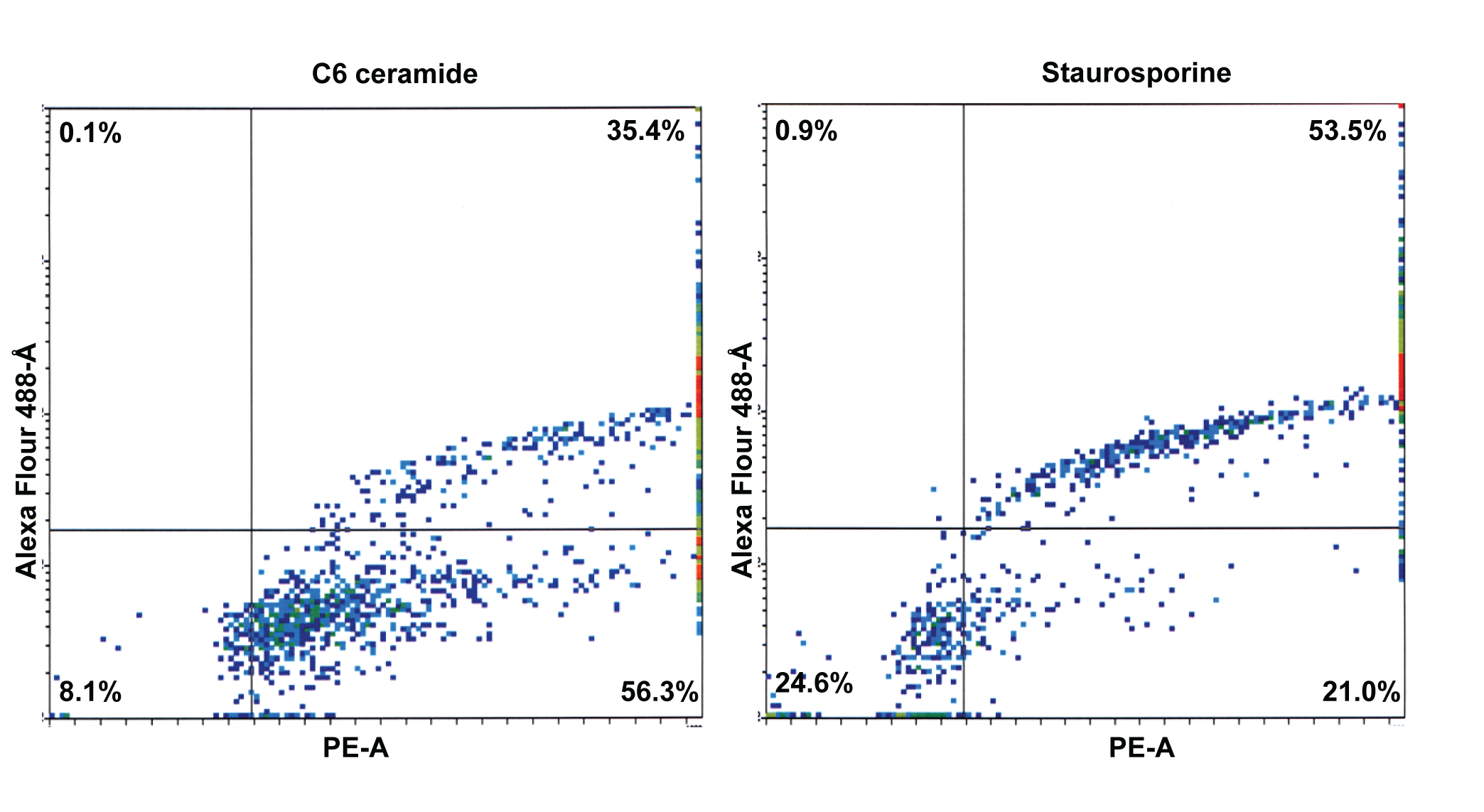

Supplement: Figure S3 — TUNEL positive cells present in cultures following C6-ceramide treatment. Cells were grown to confluence and treated overnight with 40 µM C6 ceramide (left panel), or 5 nM staurosporine (right panel). Cells were harvested and stained using TUNEL kits. The cells were analyzed by flowcytometry. Alexa Flour 488-A stained represent BrdU positive cells and PE-A stained represent propidium iodide positive cells (see File S2). (TIF) [file pone.0018137.s003.tif]

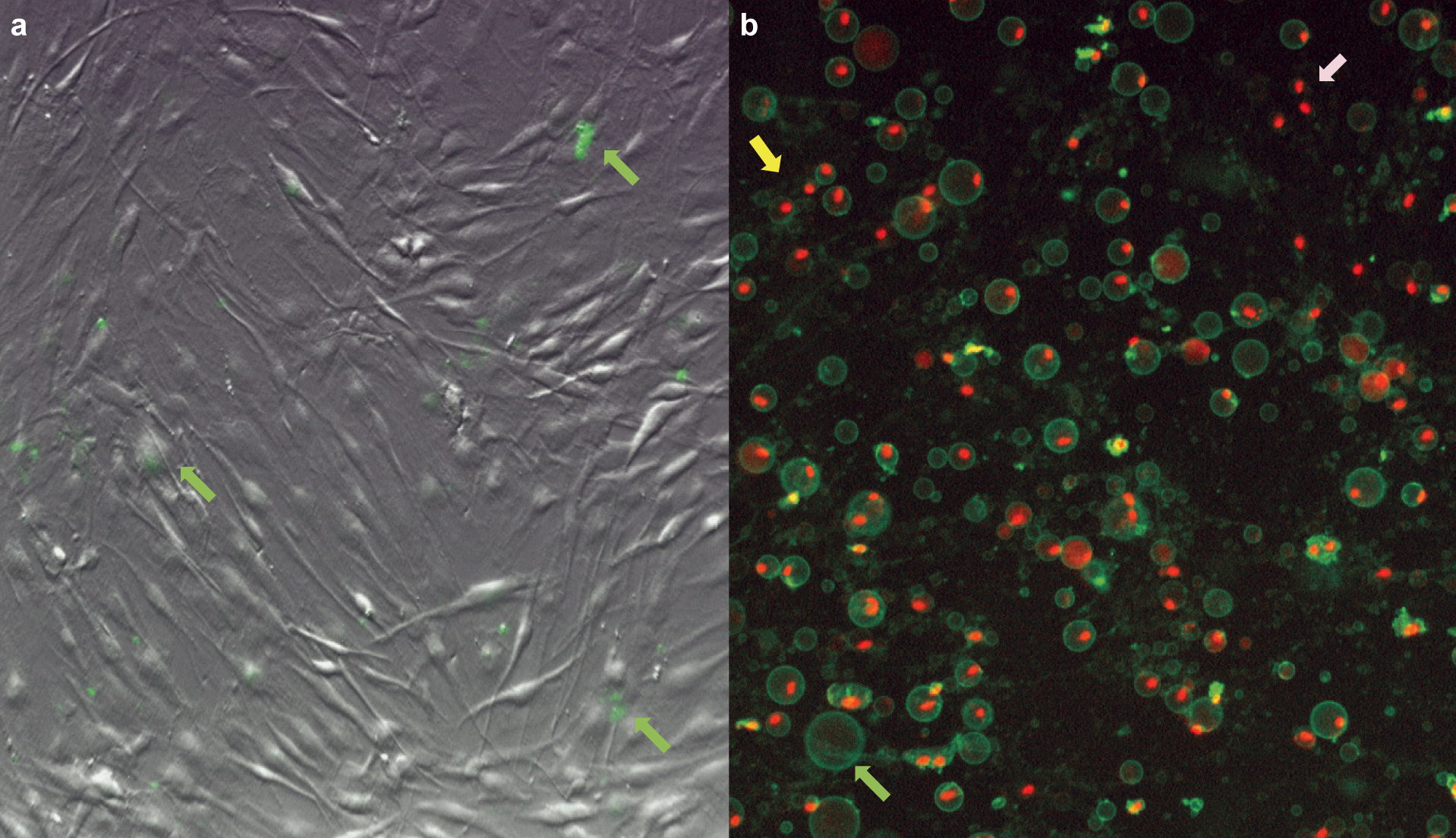

Supplement: Figure S4 — Annexin V-affinity, resulting from phosphatidylserine (PS) exposure at the outer leaflet of the plasma membrane, apoptotic cells can be distinguished from annexin V-negative living cells, by using fluorescent microscopy procedure. When combined with propidium iodide (PI) the double labeling procedure allows a further distinction of necrotic (pink arrow head, annexin V-/PI+), early apoptotic (green arrow head, annexin V+/PI-) or late apoptotic/necrotic (yellow arrow head, annexin V+/PI+) cells. The cells were used for fluorescent microscopy; the images were captured and measured for green and red fluorescence. Cells were treated with dihydroceramide (a) or 40 µM C6 ceramide (b) (see File S2). (TIF) [file pone.0018137.s004.tif]
