# Supplementary material for: Mitochondrial Dysfunction Links Ceramide Activated HRK Expression and Cell Death
Source: PLoS One. 2011 Mar 31;6(3):e18137. doi: 10.1371/journal.pone.0018137 (PMC3069046; doi:10.1371/journal.pone.0018137)
Supplement: File S1 — Reference for Figures S1 and S2. (DOC) [file pone.0018137.s005.doc]

Notredame C, Higgins DG, Heringa J (2000) T-Coffee: A novel method for fast and accurate multiple sequence alignment. J Mol Biol 302: 205-217.
