# Supplementary material for: Mitochondrial Dysfunction Links Ceramide Activated HRK Expression and Cell Death
Source: PLoS One. 2011 Mar 31;6(3):e18137. doi: 10.1371/journal.pone.0018137 (PMC3069046; doi:10.1371/journal.pone.0018137)
Supplement: File S2 — Material and Methods for Figures S3 and S4. (DOC) [file pone.0018137.s006.doc]

**Material and Methods for Supporting Figures S3 and S4**

**TUNEL Assay by flowcytometry**

The percentage of cells undergoing apoptosis was calculated as the ratio of apoptotic cells to total cells. Apoptotic HCSF detach from the substratum were collected as floaters; thus, the extent of apoptosis can be determined by counting floating and adherent cells with a FACScalibur flowcytometer (Becton-Dickinson, San Jose, CA, USA). For TUNEL assay, floating cells were recovered from the medium by centrifugation and pooled with the adherent cells collected by trypsinization and neutralized with Soybean trypsin inhibitor. Cells were spun down and TUNEL assays were performed with a commercially available kit for detecting end-labeled DNA according to the manufacturer's instructions (ApoAPO-BrdU™ TUNEL Assay Kit - with Alexa Fluor® 488 anti-BrdU, Cat # A35126, Invitrogen, Madison, WI; USA) with slight modifications. All experiments were repeated in triplicates and at least three independent occasions.

**Annexin V Staining**

HCSF were seeded in 6 wells plate at 50-60% confluency. After 48h, cells were preincubated for 12 h in the presence or absence of 40µM C6C, C6DHC and no treatment group as controls. The media was removed and cells were washed with PBS while in the wells. The cells were then incubated for 15 minutes (37˚C) in 400µl freshly prepared Annexin binding buffer (10mM HEPES, 140mM NaCl, and 2.5mM CaCl2, pH 7.4) containing 20 µl/ml Annexin V-Alexa Flour 488 conjugate (Cat #A13201, Invitrogen, Madison; USA) and 50µl/ml of Propidium iodide (P3566, Invitogen,Madison; USA ). Fluorescent microscopy was performed and images were captured.
